# Stair-Fall Risk Parameters in a Controlled Gait Laboratory Environment and Real (Domestic) Houses: A Prospective Study in Faller and Non-Faller Groups

**DOI:** 10.3390/s24020526

**Published:** 2024-01-15

**Authors:** Malarvizhi Ram, Vasilios Baltzopoulos, Andy Shaw, Costantinos N. Maganaris, Jeff Cullen, Thomas O’Brien

**Affiliations:** 1 Research to Improve Stair Climbing Safety (RISCS), School of Sport and Exercise Sciences, Faculty of Science, Liverpool John Moores University, Byrom Street, Liverpool L3 3AF, UK; 2 Faculty of Engineering and Technology, Liverpool John Moores University, Byrom Street, Liverpool L3 3AF, UK

**Keywords:** FCL—foot contact length, FCL1—foot contact length step 1, FC—foot clearance, SD—standard deviation, M—mean, Y—yes, N—no, Df—degree of freedom, Sig—significance, N—total participants, CoM—center of mass, RMP1—first metatarsophalangeal joint, RMP5—fifth metatarsophalangeal joint

## Abstract

Background: Falling on stairs is a major health hazard for older people. Risk factors for stair falls have been identified, but these are mostly examined in controlled biomechanics/gait laboratory environments, on experimental stairs with a given set of step dimensions. It remains unknown whether the conclusions drawn from these controlled environments would apply to the negotiation of other domestic staircases with different dimensions in real houses where people live. Objectives: The aim of this paper is to investigate whether selected biomechanical stepping behavior determined through stair gait parameters such as foot clearance, foot contact length and cadence are maintained when the staircase dimensions are different in real houses. Methods: Twenty-five older adults (>65 years) walked on a custom-made seven-step laboratory staircase. Older adults were classified into two groups (fallers and non-fallers) based on recent fall history. Among the 25 participants, 13 people had at least one fall, trip, or slip in the last six months and they were assigned to the fallers group; 12 people did not experience any fall in the last six months, so they were assigned to the non-fallers group. In addition, these participants walked on the stairs in three different real exemplar houses wearing a novel instrumented shoe sensor system that could measure the above stair gait parameters. MATLAB was used to extract fall risk parameters from the collected data. One-way ANOVA was used to compare fall risk parameters on the different staircases. In addition, the laboratory-based fall risk parameters were compared to those derived from the real house stairs. Results: There was a significant difference in selected stair-fall biomechanical risk factors among the house and laboratory staircases. The fall risk group comparisons suggest that high-risk fallers implemented a biomechanically riskier strategy that could increase overall falling risk. Conclusions: The significant differences due to the main effects of the fallers and non-fallers groups were obtained. For example, when ascending, the fallers group had less foot clearance on the entry (*p* = 0.016) and middle steps (*p* = 0.003); in addition, they had more foot clearance variability on the entry steps (*p* = 0.003). This suggests that the fallers group in this present study did not adopt more conservative stepping strategies during stair ascent compared to low-risk older adults. By showing less foot clearance and more variability in foot clearance, the risk for a trip would be increased.

## 1. Introduction

In daily life situations, people encounter a wide range of staircases with various dimensions, and all have a different influence on the risk of falling [1,2,3,4]. Staircase dimensions are essential to avoid stair falls, and irregular or challenging step dimensions in stairs can amplify the risk of falling [5,6]. The tread of the step (going) on the stair is vital in determining the percentage of foot length placed on a stair tread. Refs. [6,7] have shown that the gait of stair users depends on the size of the going such that if the going is large enough, users can place their whole foot onto the flat part of the tread. As the going decreases, the user allows their toes to hang over the edge of the tread or significantly turns their feet to continue their descent, which may increase the likelihood of a stairway fall. The authors of [2] have shown that people manage to put a significantly greater part of their foot on a tread for goings of 300 mm and above compared to goings of 275 mm or less. This suggests that the tread of the steps (going) on the stair is vital in determining the percentage of foot length placed on the step. The staircase risers are also very important for avoiding trips. If the risers are high, older people may become fatigued quickly and become vulnerable to trips [2]. Staircases with a large step risers create additional demands for joint moment generation during stair ascent [8] and control of the CoM during descent [9]. When risers are very short or shallow, older adults may be tempted to take more than one step at a time, which leads to more chances of mis-stepping. The steepness or pitch of a stair may also influence the likelihood of a fall.

The UK Building Regulations permit a private staircase to be made up of individual steps with each rise measuring between 100 and 220 mm, a going length between 225 and 350 mm and a maximum incline of 41.5°. Similarly, public staircases [6] must be made up of individual steps with each rise measuring between 100 and 190 mm, a going length between 250 and 350 mm and a maximum incline of 38°. These ranges permit considerable variation in staircase design.

The staircase structure can magnify the demands placed on the individual. For example, steep staircases create larger loading forces upon foot contact and cause more significant redistribution of forces at the joints than less steep staircases. It is also known that the transition steps from the level onto the stairs or from the stairs onto the level are more demanding than the continuous steps in-between [1]. More importantly, staircases with inadequate step going to safely place the foot can restrict movements and threaten safety [2,7]. 

Additionally, a stair with a higher riser is more challenging for older adults with increased muscle weakness. It has been found that older adults can safely negotiate stairs with a lower step riser compared to younger adults [10]. It is also evidenced that even for standard step risers, older adults operate closer to their maximum capacities of joint range of motion [10], thus increasing their risk for a fall. Risky techniques employed by older people during stair negotiation can also increase the risk of falling. Older people might also change their techniques over time because of their functional impairments or fear of falling [11]. For example, older adults may have a large foot overhang on landing when stair walking and higher variability in foot clearance [12], both of which increase the risk for a slip or trip on the stairs.

Identifying stair-fall risk factors is mostly limited to a controlled environment, typically in a biomechanics gait laboratory using experimental staircases with a given set of step dimensions. This is the case because biomechanical measurements of stair negotiation require specialized equipment typically present in a gait lab, including optoelectronic cameras and specially made staircases, usually of standard step dimensions, instrumented with force plates [13]. However, it remains unclear whether the conclusions drawn would still apply during stair negotiation in a real house where people live with different types of domestic staircase designs that may also have different dimensions. So, this study aims to examine older adults’ fall risk in a controlled environment (laboratory) as well as real domestic houses (LJMU’s exemplar houses). The purpose was to predict stair risk parameters and to investigate whether selected biomechanical stepping behavior determined through stair gait parameters, such as foot clearance, foot contact length ratio and cadence, was maintained when the staircase design and dimensions were different between a laboratory environment and real houses and between previous fallers and non-fallers.

## 2. Methods

### 2.1. Participants

Twenty-five older adults participated in this study (female: 20; male: 5; age: 70.72 ± 4.0 Y; body mass: 70.18 ± 10.0 kg; body height: 1.62 ± 0.06 m (mean ± standard deviation)). All the participants were recruited from the local community of Wirral and Liverpool, UK. All these participants are living independently and able to climb stairs without help. The study was approved by the Liverpool John Moores University ethics committee in the UK (REF: 18/SPS/024). After the explained procedure, informed written consent was obtained from all participants.

All the participants were recruited from the local community of Wirral and Liverpool, UK. All these participants were living independently and able to climb stairs without help. We recruited people who were living independently in a house with staircase. So, these people would need to negotiate staircases every day on their own. These were the only criteria we used. People living in bungalows were not eligible for this study, because bungalows do not have staircases. People who needed a support to climb the staircase were not eligible for this study. We made records of our participants’ previous fall history. We included both previous fallers and non-fallers. We also made records of their fear of falling on stairs. A Berg balance scale test was performed to measure participants’ stability. After data collection, participants were followed up for six months to record any falls during that time. Based on these follow-ups, participants were divided into fallers and non-fallers.

### 2.2. Staircase Configuration

**Figure 1:**
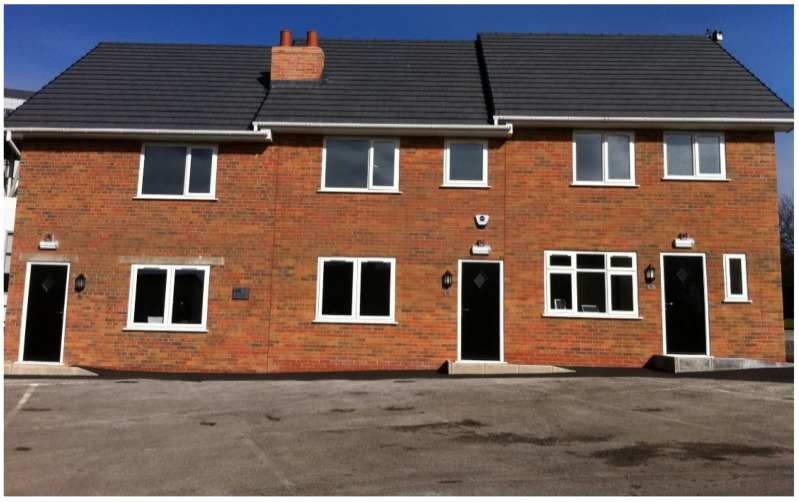
LJMU’s exemplar houses, which include three houses that are constructed to emulate domestic houses from different eras (1920s, 1970s and 2010s).

The measurements were conducted in LJMU’s exemplar houses and on a custom-built seven-step staircase in the biomechanics laboratory. Liverpool John Moores University (LJMU) has a branch of the BRE (Building Research Establishment) Innovation Park on LJMU’s Campus. The Innovation Park consists of three exemplar houses (Figure 1) that LJMU and BRE use to provide test facilities. These three houses are typical examples of domestic houses from different eras and have been constructed using staircase designs from the 1920s, 1970s and 2010s.

**Figure 2:**
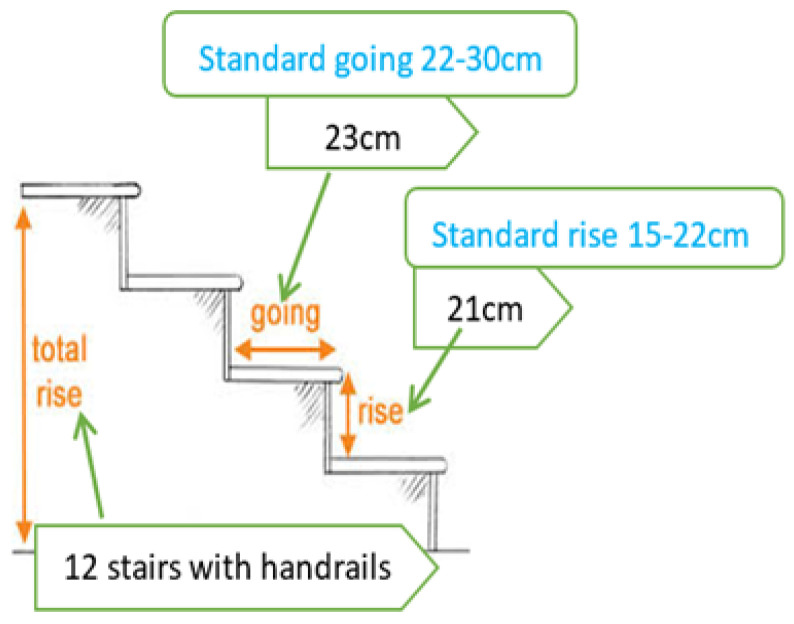
1920s and 1970s exemplar house staircase design.

The exemplar houses have three different staircases (Figure 2). Space (area) was considered an essential factor in choosing different staircases for each exemplar house. The 1920s house staircase was a straight staircase, which ran directly from the ground floor landing to the top floor landing with 12 steps and a handrail. The standard staircase going (width) was between 22 cm and 30 cm, and the standard rise (height) was 15 cm to 22 cm.

The 1920s house staircase had a 23 cm going and 21 cm rise (minimum going and nearly maximum rise). The 1970s house staircase was like the that of the 1920s, except for the staircase location (the 1970s staircase was set next to the entrance door, and the 1920s staircase was placed in the middle of the house between two rooms). The staircase comprised a single linear flight that did not change direction (Figure 2 for 1920 and 1970s house staircases).

**Figure 3:**
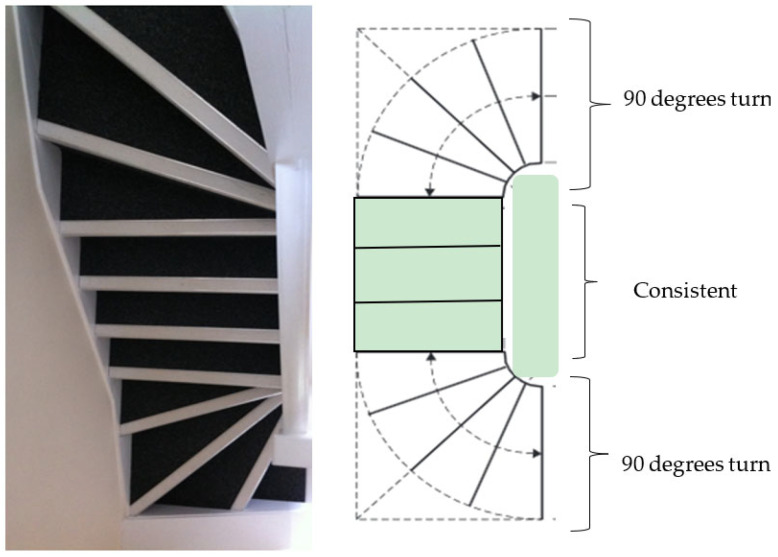
2010s staircase design in the LJMU exemplar house.

The 2010s exemplar house had a winder staircase, with 11 steps and no handrail. The winder stairs were L-shaped, but instead of a straight landing, these stairs incorporated a 90-degree turn at the start and the end of the stairs (Figure 3). The winder stairs created exciting features with a seamless transition and saved more space without a landing. However, these stairs were more challenging to navigate than the other stairs. It is also true that negotiating winder stairs requires more center support.

The winder staircase was narrower on one side than the other. A series of winder stairs form a half-circular-shaped stairway. Three steps were used to form a 90-degree turn; the intermediate step is called a kite winder as it looks like a kite-shaped quadrilateral. Figure 3 shows the 2010s exemplar house staircase.

**Figure 4:**
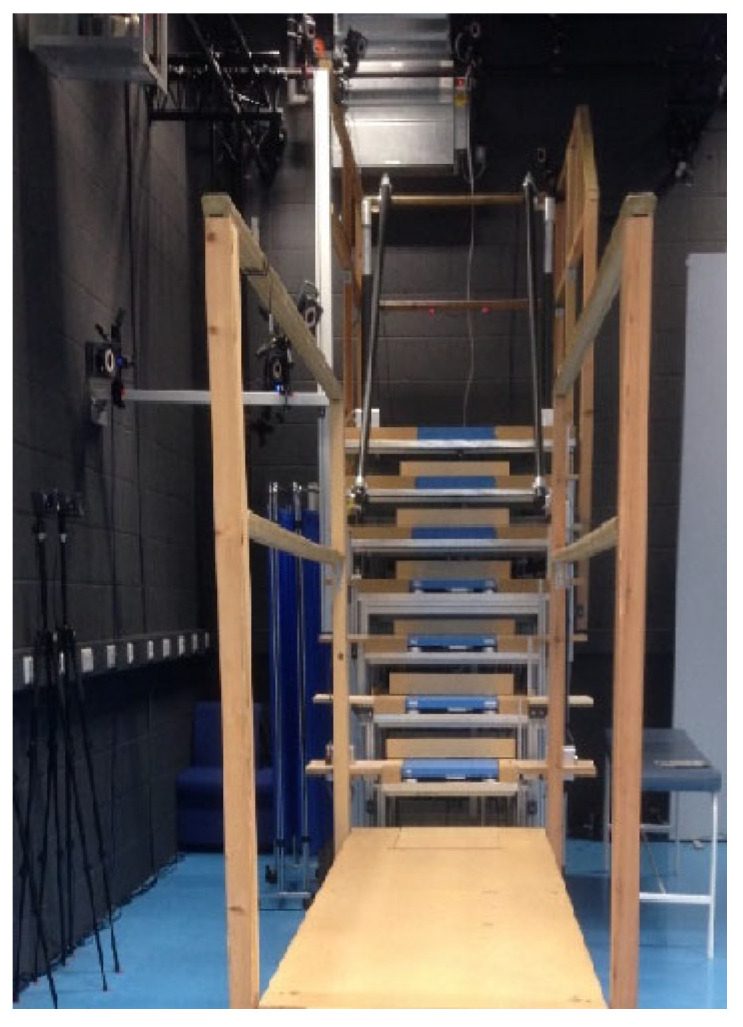
Laboratory custom-built instrumented seven-step staircase structure.

The laboratory measurements were conducted on a custom-made instrumented seven-step staircase with handrails on each side of the experimental staircase. The stairs had a top and bottom landing of sufficient length to complete an entry and exit phase. Each step had a riser height of 19.5 cm and a going length of 23.5 cm [14]. The bottom four steps contained a Kistler (9260AA, Kistler AG, CH) force platform on each step (Figure 4).

### 2.3. Procedures

Data collection took place with two sessions lasting approximately 2 h with a short break between. Special shoes instrumented with various sensors were used for data collection; the design of these instrumented shoes and the stair-fall risk parameter calculation is explained in [15] and include a novel insole sensor for estimating foot contact length and sensors to measure foot clearance. For the laboratory data, the foot clearance and percentage foot contact length and the foot’s 3D motion were captured using 24 infrared Vicon cameras, covering the whole stairs, landing, and walkway (Vicon, Oxford Metrics, UK). Kinetic data were synchronously recorded from four different force platforms (9260AA, Kistler AG, CH, Bern, Germany), embedded in the lower four steps (Steps 1–4, see Figure 4). Foot markers were placed on the lateral and medial malleolus (ankle), the first and fifth meta-phalange joints (base of big and little toe) and the posterior calcaneus (heel). Additional markers were placed on the lateral and medial calcaneus, and a rigid cluster of three markers was placed over the toes.

The first testing session was carried out in the biomechanics laboratory, and the participants completed the Berg balance assessments, as well as previous fall history and fear of falling questions. All participants were familiarized with the custom build laboratory staircase before data collection. Participants wore tight-fitting clothes and instrumented sensor shoes based on their shoe size and markers during familiarization. Participants were then fitted into the 5-point safety harness connected to an overhead safety rail via a rope, controlled by a trained member of the research team who was also secured via a cable to the floor. The participants navigated the stairs step-over-step and were permitted to use the handrails if they wished. Participants performed five more trials, with the final three trials used for data analysis.

After a break, the second testing session was undertaken at LJMU’s exemplar houses. All participants were familiarized with all three exemplar houses’ staircases before the data collection. Participants wore comfortable clothes and instrumented shoes; no markers and cameras for motion analysis were used in the exemplar houses. The sensors in the instrumented shoes and a computer were used to collect data in the houses. All participants performed three ascending and descending trials for each house (3 houses × 3 ascending × 3 descending). The participants navigated the stairs step-over-step and were permitted to use the handrails if they wished.

### 2.4. Data Analysis

The percentage of foot contact length reflects the danger of slipping and falling as a result of foot placement relative to the step edge. A lower percentage of foot contact length is linked to a higher risk of falling. Foot clearance is a measure of trip-induced fall risk, with a lower foot clearance indicating a higher chance of falling due to a trip. As a result, these parameters were examined in this study to determine the risk of falling.

The instrumented shoe was synchronized with the VICON system in the laboratory, so both shoe sensor based and VICON data were collected simultaneously. In addition, foot clearance was calculated from the distance sensor in the instrumented shoes, and the percentage of foot contact length was calculated from the FSR sensor insole. Foot clearance was calculated from the shoe sensors using the data analysis method from [15] and foot contact length from the instrumented shoe sensors was calculated using [15].

**Figure 5:**
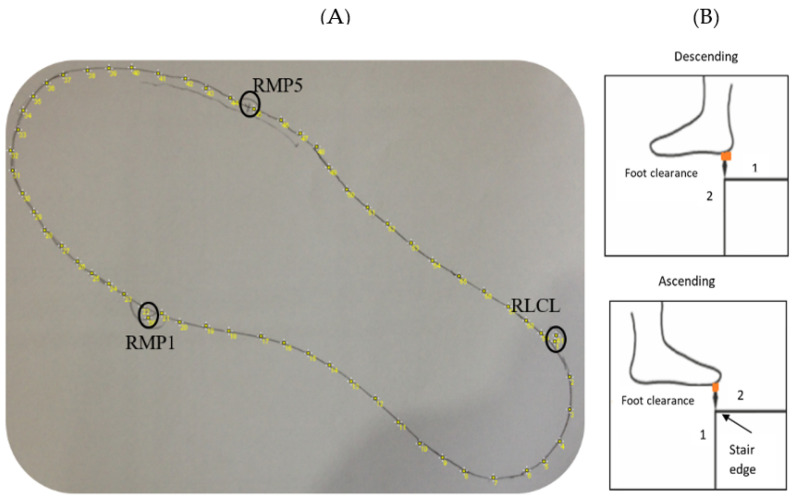
Vicon foot clearance calculation of the right foot using the above foot model. A two-dimensional outline of the shoe (**A**) was digitized and linked to three markers (first metatarsophalangeal joint: RMP1; fifth metatarsophalangeal joint: RMP5; and calcaneus lateralis: RLCL) of the static measurement. The virtual outline of the shoe was then projected in the movement trials. Foot clearance was calculated as the minimal distance between the virtual shoe and the step edge, within the orange-colored area between 1 and 2 shown in (**B**).

Foot clearance and foot contact length calculated from the VICON system motion analysis data are explained in the previous work [16,17]. In brief, the participant’s instrumented shoes were digitalized manually by obtaining a two-dimensional outline after taking a picture of the shoe outline drawn on a piece of A4 paper (Figure 5A) and imported using ImageJ 1.38e (National Institutes of Health, Bethesda, MD, USA). The coordinates of up to 600 virtual markers representing the individual shoe sole outline were calculated in MATLAB. The position of three markers fixed on the shoe were also recorded (first metatarsophalangeal joint (RMP1), fifth metatarsophalangeal joint (RMP5) and calcaneus lateral (RLCL)) in the two-dimensional drawing using the static measurement. These static measurements included the above three markers’ positions in a 3D (three-dimensional) space, which helped determine the position of the shoe’s virtual outline relative to the markers. The virtual outline of the shoe was then projected in movement trials, again relative to the three reference foot markers.

The foot clearance (Figure 5B) was obtained during the swing phase when the virtual shoe outline of the leading limb passed the vertical position (1) of the step edge up until the outline passed the horizontal position of the step edge (2). The minimal clearance of the virtual shoe was determined within this time frame for steps 1–7 in all three trials. The mean value across the three trials was considered for further analysis.

**Figure 6:**
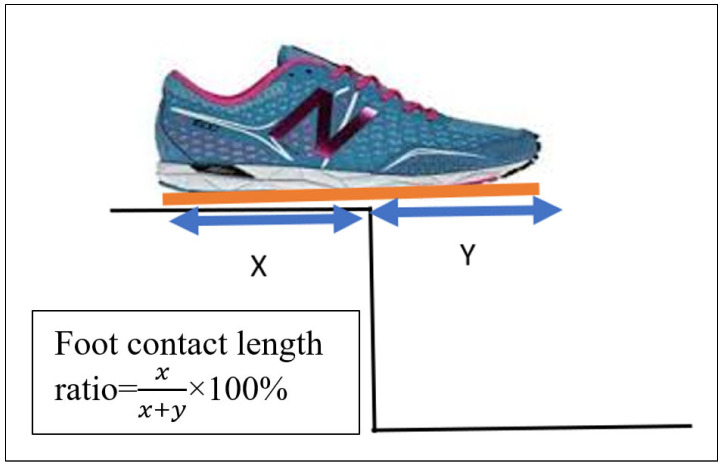
VICON foot contact length ratio calculation. The foot contact length ratio was calculated at touch-down using the rigid virtual shoe (blue line) as follows: foot placement ratio = (x/(x + y)) × 100%. Orange line shows the total length of the shoe.

We calculated the foot contact length ratio using the foot touchdown over the force plate, placed on steps 1 to 4. Distance X was measured (the distance between the step edge and the posterior foot end of the virtual shoe line), and distance Y is the distance between the step edge and the most anterior foot end of the virtual shoe outline (Figure 6). Foot contact length ratio was calculated using the formula $\frac{x}{\left(x+y\right)}\times 100\%$. The mean value across three trials was calculated and used for further analysis.

Rapidly descending the stairs can lead to a fall since the foot clearance and contact length can be affected by the increased speed. As a result, a cadence for stair ascent and stair descent was calculated using the average time of two gait cycles (one of the left limbs and one of the right limbs). The trial-to-trial variability of these parameters was determined as the average of the variability over the three trials for each of the steps, in addition to foot clearance, foot contact length, and cadence. More fluctuation can suggest a person’s inability to maintain a steady/safe movement pattern, which can increase the danger of falling [18].

### 2.5. Statistics

Following the testing, older adults were observed for 6 months and were divided into fallers and non-fallers based on whether they fell during that time. A fall was described as an accident that caused a person to fall to the ground, floor, or other lower level.

Three ANOVA comparison tests were conducted; the first ANOVA compared the difference in fall risk parameters between individuals in different houses (independent of fall history). With an Alpha level of 0.05, one-way ANOVA and post hoc tests were performed. Tukey’s HSD tests were used in post hoc analyses for multiple comparisons. Raw data from three trials for each house (18 trials for each participant, 9 trials for ascending and 9 trials for descending), a total of 25 participants (18 × 25 = 450 total trial data) and each step were treated separately, i.e., individual analyses were performed for each of the eleven steps, to compare between the houses.

The second ANOVA test was conducted to compare the difference between the results from the laboratory and houses (independent of follow-up fall details). There were seven steps in the laboratory; foot clearance was calculated for all seven steps in the laboratory. To compare this seven-step foot clearance with house data, only the foot clearance on the first, fourth, fifth, sixth, seventh, eighth and last steps were considered for houses. The foot contact length ratio was calculated for four steps where force plates were placed in the laboratory. Only the first four steps’ foot contact lengths were considered to compare this four-step foot contact length with that in a house.

The final ANOVA test looked at the differences in fall risk parameter measures between the two fall risk groups and within each condition (laboratory versus exemplar house stairs and fallers versus non-fallers). For ascent and descent, a mixed ANOVA test was used. Post hoc tests were used where necessary in the case of significant interactions. Tukey’s HSD tests were used for multiple comparisons in post hoc analyses. The Alpha level was set at 0.05. For this test, the data were averaged for all three trials; only the averaged start, end and middle stairs data were used to compare the results from the laboratory and houses.

## 3. Results

### 3.1. Differences between Three Houses’ Staircases during Stair Ascent

**Figure 7:**
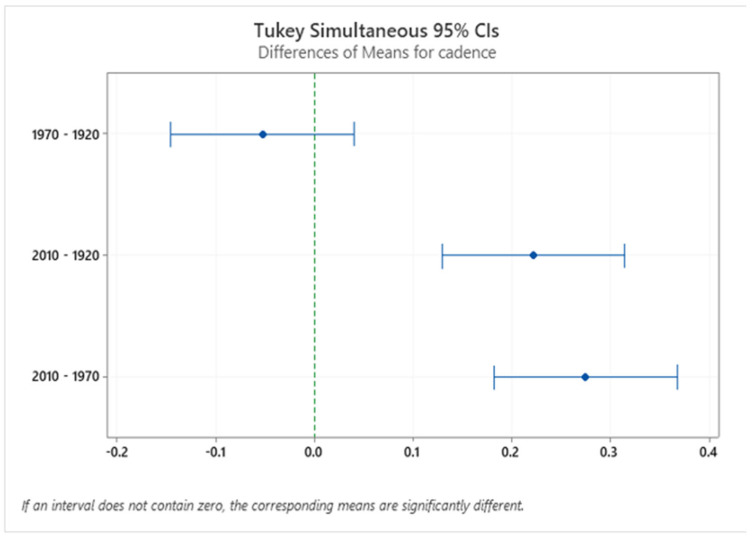
Confidence interval (CI) for cadence in the three different houses. The confidence interval range for the means of these house pairs (2010–1970, 2010–1920) does not include zero, which indicates that the difference is statistically significant.

There was a significant difference (Figure 7) in cadence between different houses’ stairs (F (2.224) = 25.52, *p* = 0.0000). Similar cadences were used in the 1920s and 1970s house stairs (M = 0.850 s, M = 0.800 s). In contrast, older adults took more time to climb the 2010s stairs due to the winder design (M = 1.08 s). These results show that older adults were more cautious while using difficult (winder) stairs.

**Figure 8:**
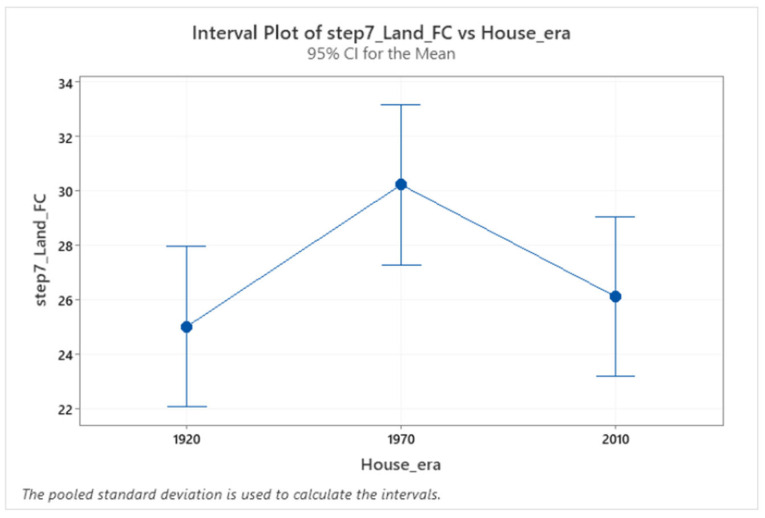
Confidence interval (CI) for foot clearance in the three different houses. The confidence interval range for means of the house pairs (1970–1920) does not include zero, which indicates that the difference is statistically significant.

Older adults’ foot clearance increased on the 1970s staircase (Figure 8). For example, there was a main effect of different staircase dimensions on foot clearance over step 7 (F (2.224) = 3.39, *p* = 0.0036). Post hoc comparisons revealed increased foot clearance on the 1970s (M = 30.23mm) compared to 1920s and 2010s (M = 25.01 mm, M = 26.12 mm) staircases. These reduced foot clearances on the 1920s and 2010s stairs might lead to an increase in the chances of falling [11]. There were no changes in foot clearance during the entry and exit steps on different houses’ staircases.

Table 1 describes in more detail the foot clearance on individual steps in the three different houses. Even though there were slight differences in the foot clearance between different houses, these differences were not statistically significant, except for step 7.

**Figure 9:**
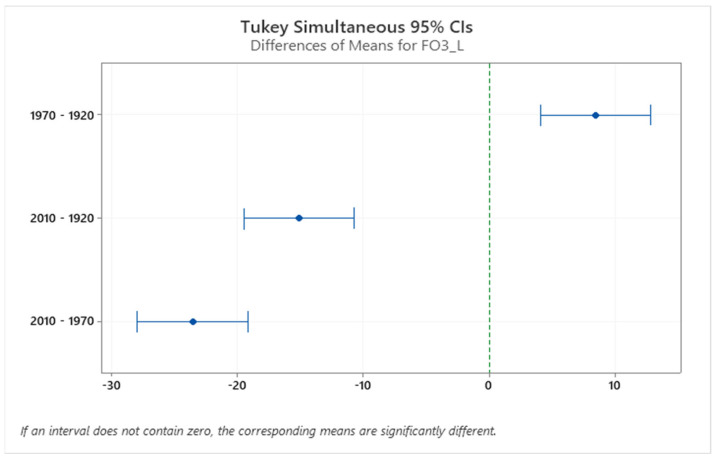
Confidence intervals (CIs) for foot contact length in the three different houses. The confidence intervals for the range of the means of all three house pairs do not include zero, which indicates that the difference is statistically significant for all three houses.

Older adults’ percentage of foot contact length decreased on the 2010s staircase due to the lower going on the stair dimension. There was a main effect of different staircase dimensions on the percentage of foot contact length over step 3 (F (2.222) = 83.127, *p* = 0.0000) and step 4 (F (2.222) = 80.8540, *p* = 0.0000). Post hoc comparisons revealed a decreased percentage of foot contact length (Figure 9) on the 2010s staircase on step 3 and step 4 (M = 59.29% on step 3, M = 59.29% on step 4) compared to the 1920s (M = 74.90% on step 3, M = 73.78% on step 4) and 1970s (M = 83.36% on step 3, M = 79.61% on step 4).

Due to the winder staircase in the 2010s house, older adults’ percentage of foot contact length significantly reduced, which may initiate a slip-induced fall. Due to the straight staircases in the 1920s and 1970s houses, the percentages of foot contact length were not significantly different, except for step 3 and step 4. However, the percentage of foot contact length is significantly different for the 2010s staircase and the other two staircases for the remaining steps. Table 2 shows the significant percentage of foot contact length among the three houses for all steps.

### 3.2. Differences between Laboratory and Houses during Stair Ascent

A second ANOVA test was conducted to compare the differences between the results from the laboratory and houses (independent of follow-up fall details). There were seven steps on the laboratory staircase, and foot clearance was calculated for all seven steps. Only the first, fourth, fifth, sixth, seventh, eighth and last steps’ foot clearance averages were considered from the houses’ staircases to compare their data with the laboratory data. For the laboratory, foot contact length was calculated for the first four steps that were instrumented with force plates. Only the foot contact lengths on the first four steps of the house staircases were considered for comparison with the laboratory (Table 3).

Older adults’ cadence decreased in the houses’ staircases compared to the laboratory. For example, there were main effects for foot cadence (F (1.98) = 8.17, *p* = 0.005). Post hoc comparisons revealed decreased cadence in the houses (mean = 0.910 s) compared to the laboratory (mean = 1.07 s) staircases. However, the ascending results showed no significant difference in cadence variability between the houses and laboratory.

Older adults’ entry steps foot clearance decreased on the houses’ staircases compared to the laboratory. For example, the main effect was on foot clearance in different environments over step 1 (F (1.98) = 5.54, *p* = 0.021). Post hoc comparisons revealed decreased foot clearance on the house staircases (mean = 26.24 mm) compared to the laboratory (M = 31.24 mm) staircase. There was no significant difference in foot clearance between the houses and laboratory during the first step on the staircases.

Older adults’ middle steps foot clearance increased on the house staircases compared to the laboratory. For example, the main effect was on foot clearance in different environments over step 2 (F (1.98) = 14.83, *p* = 0.000). Post hoc comparisons revealed increased foot clearance on the house staircases (mean = 27.66 mm) compared to the laboratory (M = 20.25 mm) staircase. Older adults’ foot clearance variability on the middle steps foot clearance variability increased on the house staircases compared to the laboratory. For example, there was a main effect for different environments’ foot clearance variability over step 2 (F (1.98) = 6.72, *p* = 0.011). Post hoc comparisons revealed increased foot clearance variability in the house staircases (mean = 8.57 mm) compared to the laboratory (M = 5.2 mm) staircase.

There were no changes in foot clearance during the exit step in different environments. However, older adults’ end steps foot clearance variability increased on house staircases compared to the laboratory. For example, there was a main effect for different environments’ foot clearance variability over step 7 (F (1.98) = 4.77, *p* = 0.030). Post hoc comparisons revealed increased foot clearance variability on the house staircases (mean = 10.35 mm) compared to the laboratory (M = 6.6 mm) staircase.

The ascending results showed no significant differences in foot contact length between the houses’ and laboratory’s first step, second step and third step. Older adults’ fourth steps foot contact length decreased on the house staircases compared to the laboratory. For example, there was a main effect of different environments on foot contact length over step 4 (F (1.98) = 5.59, *p* = 0.020). Post hoc comparisons revealed decreased foot contact length on the house staircases (mean = 70.90%) compared to the laboratory (M = 77.45) staircase. There were changes in foot contact length variability during the first, second, third and fourth steps in different environments.

### 3.3. Difference between Fallers and Non-Fallers during Stair Ascent

The third ANOVA test was conducted to compare the different environments (laboratory and houses) between the fallers group and the non-fallers group. The fallers group is considered to have a higher risk of falling due to previous fall history. On the other hand, the non-fallers group has a lower risk of falling due to a lack of previous fall history. These groups were created based on the six months of follow-up fall information.

The built environment conditions tested included four different staircases (the 1920s, 1970s, 2010s and laboratory). However, two of the staircase dimensions were the same (the 1920s and 1970s), so only one of these staircases (1920) was selected along with the 2010s house and laboratory. These selections were made was because the 1920s staircase contained consistent steps (21 cm rise, 23 cm going), and the 2010s staircase had inconsistent (winder staircase) steps. The laboratory staircase was uniform but with different dimensions (rise of 19.5 cm and going of 23.5 cm).

A mixed-model ANOVA test was performed to calculate the difference between the fallers and non-fallers groups within the different environments (three different staircase dimensions) and the interaction between the fallers and non-fallers groups and different environments. The mixed-model ANOVA results showed that the interaction between the fallers and non-fallers groups and different environments was not significant for cadence, foot clearance, foot contact length ratio (FCL) and their variability for ascending. Table 4 shows the difference between the fallers and non-fallers results in ascent.

The main effect in the fallers and non-fallers groups on entry step foot clearance variability was significant (F (1.23) = 10.613, *p* = 0.003). The post hoc results showed that non-fallers had increased entry step foot clearance variability (mean = 9.9 mm) than fallers (mean = 7.4 mm). On the other hand, the main effect in the fallers and non-fallers groups on middle and exit step foot clearance variability was not significant.

The main effect in the fallers and non-fallers groups on foot contact length was not significant. In addition, the main effect in the fallers and non-fallers groups on the entry and middle foot contact length variability was not significant.

Table 5 shows the results of the difference between fallers and non-fallers in different environments during ascent. The main effect of cadence in different environments was significant F (2.23) = 5.67, *p* = 0.000, so the mean cadence in the laboratory was significantly higher (1.069) than in the houses (1920s = 0.806 s, 2010s = 0.860 s). There was no significant difference in cadence variability in different environments.

The main effect of different environments on entry step foot clearance was significant (F (2.23) = 4.750, *p* = 0.40). The post hoc results showed that older adults had less entry step foot clearance in the houses (the 1920s = 25 mm, 2010s = 26 mm) than the laboratory (mean = 31 mm). The main effect of different environments (location) on middle step foot clearance was significant (F (2.23) = 7.663, *p* = 0.011). The post hoc results showed that older adults had less entry step foot clearance in the laboratory (mean = 20 mm) than the houses (mean 1920s = 27 mm and 2010 = 27 mm). There was no significant difference between the environments for exit step foot clearance and no significant difference between the environments for entry, middle and exit step foot clearance variability.

The main effect of different environments on entry step foot contact length (FCL) was significant (F (2.23) = 22.559, *p* = 0.000), so the entry step mean foot contact length for the 2010s house was significantly lower (67%) than for the 1920s (mean = 79%) house and laboratory (mean = 77%). Similarly, the main effect of different environments on intermediate step foot contact length (FCL) was significant (F (2.23) = 55.123, *p* = 0.000); in particular, that in the 2010s house was significantly lower (59%) than in the 1920s (mean = 74%) house and laboratory (mean = 77%).

The main effect of different environments on entry step foot contact length (FCL) variability was significant (F (2.23) = 11.945, *p* = 0.002), so the entry step mean foot contact length variability for the laboratory (mean = 3.8%) was higher than for the houses (mean = 1%). Similarly, the main effect of different environments (houses/laboratory) on middle step foot contact length (FCL) variability was significant (F (2.23) =17.603, *p* = 0.000), so the entry step mean foot contact length variability for the laboratory (mean = 2.3%) was higher than for the houses (mean = 1%).

### 3.4. Differences between Stair Descent on the Three Houses’ Staircases

The first ANOVA test for the descending results showed a significant cadence difference between the house stairs (F (2.224) = 8.249, *p* = 0.0000). A similar cadence was used on the 1920s and 2010s house stairs (M = 1.0 s, M = 1.03 s); in contrast, the older adults took more time to climb the 1970s stairs (M = 1.2). These results showed that older adults were not more cautious while using difficult (winder) stairs; going fast will lead to stair fall.

Older adults’ foot clearance was increased on the 1970s staircase. For example, there was a main effect of different staircase dimensions on foot clearance over step 10 (F (2.224) = 3.413, *p* = 0.035). Post hoc comparisons revealed increased foot clearance on the 1970s (M = 34.02 mm) compared to the 1920s and 2010s (M = 32.01 mm, M = 29.12 mm) staircases. The reduced foot clearance on these 1920s and 2010s stairs might lead to an increase in the chances of falling. There were no changes in foot clearance during the entry and exit steps on the different houses’ staircases.

Table 6 shows the percentage of foot contact length among the three houses for all steps. Older adults’ percentage of foot contact length (foot overhang) was reduced on the 2010s staircase. There was a main effect of different eras of staircases on the percentage of foot contact length from step 2 (F (2.222) = 171.447, *p* = 0.0000) to step 11 (F (2.222) = 390.811, *p* = 0.0000). Post hoc comparisons revealed a reduced percentage of foot contact length on 2010s house step 2 to step 11 (M = 65.82% on step 2, M = 69.54% on step 11) compared to the 1970s (M = 84.52% on step 2, M = 89.05% on step 11) and 1920s (M = 84.52% on step 2, M = 84.80% on step 11). Due to the winder staircase in the 2010s house, older adults’ percentage of foot contact length was significantly reduced, which might initiate a slip-induced fall. Due to the straight staircase in the 1920s and 1970s houses, the percentages of foot contact length were not significantly different.

### 3.5. Differences between Laboratory and Houses during Stair Descent

The second ANOVA test was conducted to test the differences between the laboratory and houses (independent of follow-up fall details). The descending results (Table 7) showed no significant differences in cadence and cadence variability between the houses and laboratory.

Older adults’ entry step foot clearance increased on the house staircases compared to the laboratory. For example, the main effect was on foot clearance in different environments over step 1 (F (1.98) = 22.08, *p* = 0.0000). Post hoc comparisons revealed increased foot clearance on the house staircases (mean = 28.13 mm) compared to the laboratory (M = 19.30 mm) staircase. Older adults’ entry step foot clearance variability increased on the house staircases compared to the laboratory. For example, there was a main effect for different environments’ foot clearance variability over step 1 (F (1.98) = 15.33, *p* = 0.000). Post hoc comparisons revealed increased foot clearance variability on the house staircases (mean = 7.6 mm) compared to the laboratory (M = 3.6 mm) staircase.

Older adults’ middle step foot clearance increased on the house staircases compared to the laboratory. For example, the main effect was on foot clearance in different environments over step 2 (F (1.98) = 14.22, *p* = 0.0000). Post hoc comparisons revealed increased foot clearance on the house staircases (mean = 30.70 mm) compared to the laboratory (M = 24.35 mm) staircase. Older adults’ middle step foot clearance variability increased on the house staircases compared to the laboratory. For example, there was a main effect for different environments’ foot clearance variability over step 2 (F (1.98) = 8.17, *p* = 0.005). Post hoc comparisons revealed increased foot clearance variability on the house staircases (mean = 10.82 mm) compared to the laboratory (M = 7.3 mm) staircase.

Older adults’ end step foot clearance increased on the house staircases compared to the laboratory. For example, the main effect was on foot clearance in different environments over step 7 (F (1.98) = 6.28, *p* = 0.014). Post hoc comparisons revealed increased foot clearance on the house staircases (mean = 31.71 mm) compared to the laboratory (M = 27.48 mm) staircase. There was no change in foot clearance variability during the exit step in different environments.

The descending results showed no significant differences in foot contact length and its variability between houses and the laboratory’s first step. Older adults’ middle step foot contact length increased on the house staircases compared to the laboratory. For example, different environments had a main effect on foot contact length over step 2 (F (1.98) = 11.28, *p* = 0.001). Post hoc comparisons revealed increased foot contact length in the house staircases (mean = 89.36%) compared to the laboratory (M = 85.71%) staircase. There was no change in foot contact length variability during the second step in different environments. Also, there were no changes in foot contact length and its variability during the third step in different environments.

Older adults’ fourth step foot contact length increased on the house staircases compared to the laboratory. For example, the different environments had a main effect on foot contact length over step 4 (F (1.98) = 14.03, *p* = 0.000). Post hoc comparisons revealed increased foot contact length on the house staircases (mean = 89.42%) compared to the laboratory (M = 85.20%) staircase. There were no changes in foot contact length variability during the fourth step in different environments.

### 3.6. Differences between Fallers and Non-Fallers during Stair Descent

The third ANOVA test was conducted to compare differences in the laboratory and house stairs between the fallers and non-fallers groups when descending. A mixed-model ANOVA test was performed to calculate the difference between the fallers and non-fallers groups within the different environments and the interaction between the fall risk groups and different environments when descending.

The mixed-model ANOVA results showed that the interaction between the fallers and non-fallers groups and different environments was not significant for cadence, foot clearance, foot contact length (FCL) and their variability for descending.

Table 8 shows the differences between fallers and non-fallers when descending. The mixed-mode ANOVA test revealed that the main effect of cadence and its variability in the fallers and non-fallers groups was insignificant. The main effect in the fallers and non-fallers groups on entry, middle and exit step foot clearance and their variability was not significant. The main effect in the fallers and non-fallers groups on foot contact length and its variability were not significant.

Table 9 shows the difference between fallers and non-fallers in different environments when descending. The main effect of cadence in different environments was significant (F (2.23) = 6.788, *p* = 0.016), so the mean cadence for the 2010s house was significantly higher (1.03 s) than the mean cadence time for 1920s house (mean = 0.941 s) and laboratory (mean = 0.941 s). There was no significant difference in cadence variability in the different environments.

The main effect on entry step foot clearance in different environments was significant (F (2.23) = 15.098, *p* = 0.001); the post hoc results showed that older adults had less entry step foot clearance in the laboratory (mean = 18 mm) than in the houses (1920s = 28 mm, 2010s = 27 mm). The main effect of environments on entry step foot clearance variability was significant (F (2.23) = 8.094, *p* = 0.009); the post hoc results showed that older adults had increased variability in the houses (7 mm) than in the laboratory (mean = 3 mm).

There was no significant difference between the environments for middle step foot clearance. The main effect of environments on middle step foot clearance variability was significant (F (2.23) = 8.638, *p* = 0.007), and the post hoc results showed that older adults had increased intermediate step foot clearance variability in the houses (14 mm) compared to the laboratory (mean = 9 mm). There was no significant difference between the environments for exit step foot clearance and its variability. The main effect of different environments (houses/laboratory) on foot contact length (FCL) and its variability were not significant.

## 4. Discussion

The safety of stair negotiation depends on the interactions between the behavior of humans and their staircase environment. All older adults used the step-over-step method to negotiate the different staircases during the data collection in the houses and laboratory. This step-over-step method requires alternation between limbs, with each limb contributing to single-limb support. This method is most demanding even though it is the fastest and most efficient.

### 4.1. Comparison of Risk Factors between Different Exemplar Houses’ Staircases

The statistical analysis revealed significant differences in foot clearance, foot contact length ratio and cadence between the staircases in the different houses.

Older adults were tested on three exemplar houses’ staircases; two were straight, whereas the third staircase design and dimensions in the 2010s exemplar house differed from the other two older house designs. The reason for testing stair negotiation in three different exemplar houses is that people live in different houses built over different periods, and thus, encounter a wide variety of staircases in real life. We wanted to discover how older adults negotiate different staircase designs and dimensions and which staircase might pose a higher risk for older people. When older adults encounter staircases of different dimensions, they often change their walking trajectory to cope with that staircase. The results showed that older adults’ feet followed a similar trajectory for entry and exit and, except for a few steps, the middle of the staircase. For example, in both ascending and descending, on similar staircases (the 1920s and 1970s), older adults showed no significant statistical difference in foot contact length ratio (FCL), so their feet followed a similar trajectory for both similarly designed staircases. Also, for similar staircases (the 1920s and 1970s), older adults used similar cadences for ascending and different cadences for descending. There was no significant difference for entry and exit step foot clearance on similar staircases (the 1920s and 1970s) for both ascent and descent. However, when ascending, there was a significant difference in step 7 foot clearance on similar staircases (the 1920s and 1970s), and when descending, there was a significant difference over step 10.

When ascending, older adults spent less time climbing consistent (the 1920s and 1970s) straight stairs. Previous research [19] found that straight flights of stairs without landings accounted for 52% of all accidents. This might be the case because the path of a straight flight of stairs is often clear and uninterrupted, so stair users are reassured into a false sense of security and reduced attention. Straight flights may also result in more severe injuries because there is no place where the fall may be broken on the stairway. When ascending, older adults spent more time climbing uneven stairs (the 2010s); in contrast, when descending, older adults spent less time negotiating uneven stairs. Older adults negotiated stairs considerably faster, which is considered riskier.

The foot contact length ratio was lower (mean = 67%) for inconsistent (2010s) stairs for both ascending and descending. This shows that the risk of overstepping increases on narrower stairs (2010s) due to lack of space to place the foot safely [7]. The foot contact length ratio is more crucial for descending than ascending for safe stair negotiation [20]. For example, older adults, who usually have a lower foot contact length ratio, might experience a fall [7]. If less than 70% of the foot length has contact with the surface of the step regularly, there is an increased risk of a slip over the step-edge [21], but the British Standards Institution (BSI) indicates that a less than 50% foot contact length ratio would most likely lead to a fall (BSI 2010).

For both ascending and descending in older adults, foot clearances over the intermediate steps were reduced for the 2010s staircase, increasing the risk of a toe-catch and fall due to tripping. When individuals have less foot clearance, the chances of tripping increase [12,22].

### 4.2. Comparison of Risk Factors between Houses and Laboratory Staircases

The statistical analysis conducted for the comparison of stair-fall risk factors such as cadence, foot clearance and foot contact length ratio between house (uncontrolled environment) and laboratory stairs (controlled environment) showed significant differences for all these factors.

In ascending, older adults walked more slowly in the lab than in the houses. The measurements of this study were conducted on an experimental staircase using a safety harness in a laboratory environment, which differs from house staircases, and this might have had psychological and behavioral effects on the stair performance of the older adults’ cadence. The exemplar home staircase experiments did not use a safety harnesses, as these are built as normal domestic living environments.

Older adults showed a safe strategy for ascending in the lab; for example, they had increased foot clearance on the start and middle steps and showed less foot clearance variability. In addition, older adults’ foot contact length increased on the laboratory’s exit steps. However, older adults’ foot contact length variability was higher for the laboratory than the houses’ stairs.

In contrast, older adults showed a risky strategy while descending the laboratory stairs; for example, start, middle and end foot clearance was lower in the lab than in the houses. In addition, the intermediate step foot contact length was lower in the lab. However, the older adults also exhibited a safe technique, as there was less variability in the foot clearance on the laboratory’s entry and intermediate steps.

Older adults displayed a risky strategy for ascending in the houses; for example, they had decreased foot clearance on the start and middle steps and showed more foot clearance variability. In addition, older adults’ foot contact length decreased on the houses’ exit steps. However, foot contact length variability was lower on the staircases of the houses.

In contrast, older adults showed a safe strategy while descending houses’ stairs; for example, start, middle and end step foot clearance increased. Also, the intermediate step foot contact length increased in the houses. However, they showed more variability in foot clearance on the houses’ entry and intermediate steps.

### 4.3. Comparison of Fallers and Non-Fallers

The third ANOVA test compared differences in the stair-fall risk factors within the laboratory and houses (different environments) between the fallers and non-fallers groups. There were significant differences in cadence, foot clearance, foot contact length and their variability due to the main effect of the fallers and non-fallers groups. There were also significant differences in cadence, foot clearance, foot contact length and their variability due to the main effect of different environments (laboratory and houses staircases). Finally, there were significant differences in cadence, foot clearance, foot contact length and their variability due to the main effect of the interaction between the risk group and different environments. More specifically, there was a significant change only due to the risk group and different environments. There was no significant difference between the fallers and non-fallers groups and different environments interaction.

The mixed-model ANOVA results showed that the risk group x different environments interaction was not significant for cadence, foot clearance, foot contact length (FCL) and their variability for descending. Differences in behavior were observed between the fallers and non-fallers groups, and the effect of the different environments were similar for the fallers and non-fallers group older adults (risk group x different environment interactions were not detected). Therefore, it is expected that both groups would be at an increased fall risk via the same mechanisms on different environments’ staircases. However, the consequences will likely be more severe for the fallers group [23] as they do not have the adequate strength reserves to recover when they lose balance.

There were significant differences due to the main effects in fallers and non-fallers groups. For example, when ascending, older adults had less foot clearance on the entry and middle steps; they had more foot clearance variability on the entry steps. This suggests that the fallers group in this study did not adopt more conservative stepping strategies during stair ascent compared to older adults with lower fall risk. Showing less foot clearance and more variability in foot clearance would increase the risk for a trip [1].

Concerning differences due to the different environments, the cadence results showed that older adults ascended the laboratory staircase more slowly compared to the houses’ staircases. One probable reason for this is that the safety harness that was used in the laboratory might have affected the older adults’ cadence. Also, the results showed that older adults descended the 2010s (winder) staircase slowly (longer time) and spent less time negotiating consistent staircases such as 1920s and the laboratory stairs. Even though the winder staircase was narrow and steeper, older adults took more care when walking on the winder staircase (uneven dimensions), showing that they were more cautious on the winder staircase.

When ascending, older adults had less entry step foot clearance on the house staircases such as the 2010s and 1920s. Also, during descending, older adults showed more variability in foot clearance for entry steps and less entry step foot clearance in the laboratory. The reason for this reduced foot clearance and its variability on the entry steps have already been demonstrated; a disproportionate amount of stairway accidents occurs on the top or bottom stairs [24]. In these locations, the older adult might be looking around for the next part of the journey or the route to be taken, so their attention might not be entirely focused on the stairway [25].

When ascending, older adults showed a reduced foot contact length ratio for the 2010s (winder) staircase, and foot contact length variability increased in the laboratory. This reduced foot contact length ratio increased the risk of slipping. The reason for this might be that older adults receive their best support when they place most of their foot on the tread, but this is not always possible because the going of the winder staircase was less (below 250mm) than their foot length. To safely negotiate this small going, older adults need to turn their feet to the side on each step.

### 4.4. Other Fall Risk Parameters

The Berg balance scale (BBS) (Berg et al., 1989) was used to measure older people’s balance. The Berg balance scale contains fourteen assessment tasks such as standing with eyes closed, turning around, and standing on one leg. This Berg balance scale task assessment is subjective and qualitative, typically using threshold assessment scores to categorize people as having a low fall risk, moderate fall risk or high fall risk. The possible scores are from 0 to 56, and the maximum score of 41 to 56 signifies no balance impairment (low risk), a score of 0–20 implies a high risk of fall, and from 21 to 40 indicates a medium risk of fall. The BBS is highly sensitive and specific for identifying older adults at higher risk of falling [26]. In this study, there were 25 participants assessed based on the Berg balance scale; only 1 participant had a high fall risk, 6 participants had a moderate fall risk and 18 participants had a low fall risk. When descending, Berg balance high-risk older adults had increased cadence (mean = 1.37 s) compared to low- and moderate-risk older adults (mean = 0.810 s for low risk and mean = 0.900 s for medium risk).

Fear of falling and previous fall history was assessed via oral interview. Among the 25 participants, 8 people had a fear of falling, and 17 people did not have a fear of falling. Among these eight (fear of falling) older adults, five of them had a fall in the six-month follow-up time.

The risk of a fall is higher in older adults who have experienced a previous fall [12,27]. The older adults who had had a previous fall showed high variability in foot clearance compared to the older adults who had not had any previous falls (when ascending, previous faller: standard deviation (SD) = 8 mm vs. previous non-faller: SD = 5 mm foot clearance variability). In addition to foot clearance variability, older adults who had experienced a previous fall showed a higher percentage of foot contact length variability compared to older adults who had not had any previous falls (when ascending, previous faller: SD = 3.10% vs. previous non-faller: SD = 1.10% foot contact length variability).

Fear of falling is known as a risk factor for trips on stairs. Older adults who had a fear of falling showed increased cadence variability compared to older adults who did not have a fear of falling. Cadence variability impacts the older adult’s stability, and this reduced stability may lead to future falls.

## 5. Conclusions

There was a significant difference in selected stair-fall biomechanical risk factors among the house and laboratory staircases. Even though the 1920s and 1970s staircases had similar dimensions, older adults negotiated middle steps differently, and there were no changes in stair negotiation for the entry and exit steps. Although it has been generally considered that winder stairs are more dangerous than standard stair designs because of the non-uniform tread width or the wedge shape of the winder tread, recent studies concerning stair accidents reveal that this is not true [28]. Older adults used increased cadence and more foot clearance compared to those used in the other houses, in agreement with this observation. In contrast, the percentage of foot contact length decreased compared to that in the other houses. This is because the walking portion of the tread was less than that in the other two houses. Older adults showed a safer strategy for ascending in the laboratory and descending in the houses. In contrast, older adults showed a riskier strategy for descending in the laboratory and ascending in the houses. The fall risk group comparisons suggest that high-risk fallers implemented a biomechanically riskier strategy that could increase overall falling risk.

However, data were collected from only 25 participants; a larger sample size would be preferable. This project only focuses on different stair dimensions and how older people perform on these staircases. It would be better if we could include various risk factors such as light, carpet, etc. In the future, we need to extract more fall risk parameters within the user environment, as this would be more beneficial for predicting stair falls and implementing preventative interventions to reduce future stair falls.

This work’s approach of testing on different houses’ staircases has policy implications. It may lead to revisions of the current building regulations relating to stair design; older adults can negotiate standard stair configurations, although they adopt different strategies. However, these strategies become more common and exaggerated as the staircase configuration becomes challenging. In terms of stair design, a higher step riser imposes the highest demand on older individuals. Therefore, optimizing the step riser and step going may reduce lower-limb muscle strength demands and potentially lower fall risk.

## Figures and Tables

**Table 1:** Mean foot clearance (mm) for all three houses (ascending).

Foot Clearance	Step 1	Step 2	Step 3	Step 4	Step 5	Step 6	Step 7	Step 8	Step 9	Step 10	Step 11
1920 Mean ± SD	26.7 ± 13.5	27.9 ± 17.5	26.2 ± 11.4	27.9 ± 13.2	27.6 ± 14.4	28.3 ± 14.3	25.0 ± 11.1	29.3 ± 14.5	25.4 ± 11.7	30.5 ± 12.5	27.8 ± 13.8
1970 Mean ± SD	29.0 ± 14.1	26.4 ± 13.5	26.8 ± 12.2	28.8 ± 13.3	27.4 ± 12.5	26.7 ± 12.5	30.2 ± 14.1	27.4 ± 12.6	26.3 ± 11.5	27.0 ± 12.3	27.9 ± 13.7
2010 Mean ± SD	26.8 ± 13.4	26.1 ± 13.1	28.2 ± 14.4	27.7 ± 14.4	29.1 ± 14.0	26.9 ± 11.7	26.1 ± 13.3	29.2 ± 14.6	28.3 ± 12.8	27.8 ± 13.1	30.1 ± 14.5

**Table 2:** Post hoc results for foot contact length (FCL) ratio for each step in all three houses (ascending). FCL1 indicates foot contact length on step 1. There is a statistically significant difference in foot contact length between different houses in each step; for example, a significant difference of 1,2 indicates that the 2010s house foot contact length is different from that of the 1970s and 1920s.

Foot Contact Length Ratio N = 75	2010	1970	1920	Sig.	Degree of Freedom Df (2.222)	Significant Difference
Subset for Alpha = 0.05	Mean (%)	Mean (%)	Mean (%)		F	1 = 2010 2 = 1970, 1920 3 = 1920
**FCL1**	67.0133	78.3467	79.2000	0.000	35.7710	1, 2
**FCL2**	62.0133	75.9867	79.3600	0.000	64.2140	1, 2
**FCL3**	59.8000	74.9067	83.3600	0.000	83.1270	1, 2, 3
**FCL4**	59.2933	73.7867	79.6133	0.001	80.8540	1, 2, 3
**FCL5**	61.3067	74.4533	75.1600	0.000	32.4600	1, 2
**FCL6**	59.8533	73.3067	74.2400	0.000	51.2870	1, 2
**FCL7**	62.1867	74.5467	74.5467	0.000	26.5450	1, 2
**FCL8**	59.4133	72.9200	73.7067	0.000	52.5330	1, 2
**FCL9**	62.6933	74.3733	76.8933	0.000	31.8210	1, 2
**FCL10**	60.4267	73.0533	74.5600	0.000	46.3970	1, 2
**FCL11**	65.3867	79.7200	82.0933	0.000	61.6030	1, 2

**Table 3:** Difference between laboratory and house ascending ANOVA results. The Alpha column (Y, N) shows whether there a difference (Y) or not (N) between the house and laboratory staircases; the Alpha column (F (1.98) = 8.17, *p* = 0.005) shows that 1 is the between-groups degree of freedom and 98 is the within-groups degree of freedom (separated by a comma), with F statistics and *p* value.

Variables—Ascent	Alpha < 0.05	House (1) Mean ± SD	Laboratory (2) Mean ± SD
Cadence	Y, F (1.98) = 8.17, *p* = 0.005	0.910 ± 0.22	1.07 ± 0.25
Cadence variability	N		
Foot clearance start	Y, F (1.98) = 5.54, *p* = 0.021	26.24 ± 9.46	31.24 ± 8.37
Foot clearance middle (2)	Y, F (1.98) = 14.83, *p* = 0.000	27.66 + 9.10	20.25 ± 5.24
Foot clearance middle (3)	N		
Foot clearance middle (4)	Y, F (1.98) = 9.90, *p* = 0.002	27.56 ± 8.93	21.12 ± 8.66
Foot clearance middle (5)	Y, F (1.98) = 7.18, *p* = 0.009	26.04 ± 6.90	21.51 ± 8.56
Foot clearance middle (6)	Y, F (1.98) = 18.48, *p* = 0.000	28.40 ± 9.11	19.60 ± 8.07
Foot clearance end	N		
Foot clearance start var	N		
Foot clearance middle2_var	Y, F (1.98) = 6.72, *p* = 0.011	8.57 ± 6.11	5.2 ± 2.9
Foot clearance middle3_var	N		
Foot clearance middle4_var	N		
Foot clearance middle5_var	Y, F (1.98) = 8.10, *p* = 0.005	9.23 ± 7.4	4.90 ± 2.1
Foot clearance middle6_var	Y, F (1.98) = 4.30, *p* = 0.041	8.46 ± 6.56	5.6 ± 3.2
Foot clearance end var	Y, F (1.98) = 4.77, *p* = 0.030	10.35 ± 7.8	6.6 ± 4.8
Foot contact length start	N		
Foot contact length second	N		
Foot contact length third	N		
Foot contact length fourth	Y, F (1.98) = 5.59, *p* = 0.020	70.90 ± 11.92	77.45 ± 12.28
Foot contact length start var	Y, F (1.98) = 51.15, *p* = 0.000	0.11 ± 0.10	3.80 ± 4.34
Foot contact length second var	Y, F (1.98) = 58.00, *p* = 0.000	0.15 ± 0.17	3.52 ± 3.86
Foot contact length third var	Y, F (1.98) = 36.29, *p* = 0.000	0.097 ± 0.08	2.35 ± 3.27
Foot contact length fourth var	Y, F (1.98) = 51.53, *p* = 0.000	0.09 ± 0.08	3.0 ± 3.6

**Table 4:** Difference between fallers and non-fallers in ascent. The Alpha column (F (1.23) = 6.795, *p* = 0.016) shows that 1 is the between-groups degree of freedom and 23 is the within-groups degree of freedom (separated by a comma) with F statistics and *p* value.

Risk Parameters	Alpha *p* < 0.05	Fallers	Non-Fallers
Entry steps foot clearance	F (1.23) = 6.795, *p* = 0.016	24.77 mm	30.45 mm
Middle steps foot clearance	F (1.23) = 10.613, *p* = 0.003	22.02 mm	28.09 mm
Exit steps foot clearance	Not significant	No	No
Entry steps foot clearance variability	F (1.23) = 10.613, *p* = 0.003	7.4 mm	9.9 mm
Foot contact length and variability	Not significant	No	No

**Table 5:** Difference between fallers and non-fallers in different environments during ascent. The Alpha column (Y) shows if there a difference between houses and laboratory. The Alpha column (F (2.23) =5.67, *p* = 0.000) shows that 2 is the between-groups degree of freedom and 23 is the within-groups degree of freedom (separated by a comma) with F statistics and *p* value.

Variables—Ascent	Alpha < 0.05	Laboratory	1920	2010
Cadence	Y, F (2.23) = 5.67, *p* = 0.000	0.069 s	0.806 s	0.860 s
Cadence variability	Not significant			
Foot clearances entry steps	Y, F (2.23) = 4.750, *p* = 0.40	31 mm	25 mm	26 mm
Foot clearance middle steps	Y, F (2.23) = 7.663, *p* = 0.011	20 mm	27 mm	27 mm
Foot clearances exit steps	Not significant			
Foot clearance variability	Not significant			
Foot contact length ratio on entry steps	Y, F (2.23) = 22.559, *p* = 0.000	77%	79%	67%
Foot contact length ratio on Middle steps	Y, F (2.23) = 55.123, *p* = 0.000	77%	74%	59%
Foot contact length ratio on exit steps	Not significant			
Foot contact length ratio variability on entry steps	Y, F (2.23) = 11.945, *p* = 0.002	3.8%	1%	1%
Foot contact length ratio variability on middle steps	Y, F (2.23) = 17.603, *p* = 0.000	2.3%	1%	1%
Foot contact length ratio variability on exit steps	Not significant			

**Table 6:** Post hoc result for foot contact length ratio for each step in all three houses (descending).

Post Hoc Result for Foot Contact Length Ratio for Descent	2010	1970	1920	Sig.	Degree of Freedom (2222)
N = 75	Mean (M)	Mean (M)	Mean (M)	Alpha = 0.05	F
**Foot contact length step 1**	70.3733	84.5200	84.2533	0.0000	334.156
**Foot contact length step 2**	65.8267	84.5200	81.4267	0.0000	171.447
**Foot contact length step 3**	69.9200	89.3467	76.5067	0.0000	247.767
**Foot contact length step 4**	65.6000	86.1200	81.2267	0.0010	174.200
**Foot contact length step 5**	68.9733	89.6000	84.4633	0.0000	464.929
**Foot contact length step 6**	64.9067	84.4533	82.0000	0.0000	177.450
**Foot contact length step 7**	69.0533	89.1467	84.7600	0.0000	436.971
**Foot contact length step 8**	65.1233	86.3467	81.7733	0.0000	188.358
**Foot contact length step 9**	68.9333	89.0400	84.7867	0.0000	450.253
**Foot contact length step 10**	65.5733	85.8133	81.4667	0.0000	178.279
**Foot contact length step 11**	69.5467	89.0533	84.8000	0.0000	390.811

**Table 7:** Difference between laboratory and house descent ANOVA results. Alpha column (Y or N) shows whether there is a difference between house and laboratory. If there is a difference, then it is represented as a Y; if not, then it is represented as N. And the Alpha column (F (1.98) = 22.08, *p* = 0.0000) shows that 1 is the between-groups degree of freedom and 98 is the within-groups degree of freedom (separated by a comma) with F statistics and *p* value.

Variables for Descent	Alpha < 0.05	House Mean ± SD	Laboratory Mean ± SD
Cadence	N		
Cadence variability	N		
FC start	Y, F (1.98) = 22.08, *p* = 0.0000	28.13 ± 9.24	19.30 ± 2.58
FC middle (2)	Y, F (1.98) = 14.22, *p* = 0.0000	30.70 + 7.62	24.35 ± 6.21
FC middle (3)	N		
FC middle (4)	Y, F (1.98) = 9.93, *p* = 0.002	34.42 ± 8.51	28.33 ± 7.89
FC middle (5)	Y, F (1.98) = 5.87, *p* = 0.017	31.32 ± 6.94	27.55 ± 6.12
FC middle (6)	N		
FC end	Y, F (1.98) = 6.28, *p* = 0.014	31.71 ± 7.9	27.48 ± 5.05
FC start var	Y, F (1.98) = 15.33, *p* = 0.000	7.6 ± 4.7	3.6 ± 3.05
FC middle_var2	Y, F (1.98) = 8.17, *p* = 0.005	10.82 ± 5.3	7.3 ± 5.14
FC middle_var3	Y, F (1.98) = 7.52, *p* = 0.007	11.21 ± 5.6	7.76 ± 4.8
FC middle_var4	Y, F (1.98) = 6.14, *p* = 0.015	11.91 ± 5.5	8.8 ± 4.9
FC middle_var5	Y, F (1.98) = 4, *p* = 0.048	12.32 ± 6.0	9.54 ± 5.91
FC middle_var6	N		
FC end var	N		
FCL start	N		
FCL middle2	Y, F (1.98) = 11.28, *p* = 0.001	89.36 ± 3.52	85.71 ± 7.25
FCL middle3	N		
FCL end	Y, F (1.98) = 14.03, *p* = 0.000	89.42 ± 3.86	85.20 ± 7.15
FCL start var	N		
FCL middle_var2	N		
FCL middle_var3	N		
FCL end var	N		

**Table 8:** Difference between fallers and non-fallers when descending.

Risk Parameters for Stair Descent	Alpha *p* < 0.05	Fallers	Non-Fallers
Cadence and its variability	Not significant	No	No
Foot clearance and its variability	Not significant	No	No
Foot contact length and variability	Not significant	No	No

**Table 9:** Differences between fallers and non-fallers in different environments when descending. Alpha column (Y or Not significant) shows whether there is a difference between house (1920 and 2010) and laboratory. If there is a difference, then it is represented as a Y; if not, then it is represented as Not significant. And the Alpha column (F (2.23) = 6.788, *p* = 0.016) shows that 2 is the between-groups degree of freedom and 23 is the within-groups degree of freedom (separated by a comma) with F statistics and *p* value.

Variables—Descent	Alpha < 0.05	Laboratory	1920	2010
Cadence	Y, F (2.23) = 6.788, *p* = 0.016	0.941 s	0.941 s	1.035 s
Cadence variability	Not significant			
Foot clearance entry steps	F (2.23) = 15.098, *p* = 0.001	18 mm	28 mm	27 mm
Foot clearance middle steps	Not significant			
Foot clearance exit steps	Not significant			
Foot clearance variability on entry steps	F (2.23) = 8.094, *p* = 0.009	3 mm	7 mm	7 mm
Foot clearance variability on middle steps	F (2.23) = 8.638, *p* = 0.007	9 mm	14 mm	14 mm
Foot contact length ratio and its variability	Not significant			
Foot contact length ratio on exit steps	Not significant			

## Data Availability

The data presented in this study are available on request from the corresponding author.
